# Pharmacological interventions for sleepiness and sleep disturbances caused by shift work

**DOI:** 10.1590/1516-3180.20151331T1

**Published:** 2014-11-28

**Authors:** Juha Liira, Jos H. Verbeek, Giovanni Costa, Tim R. Driscoll, Mikael Sallinen, Leena K. Isotalo, Jani H. Ruotsalainen

## Abstract

**BACKGROUND:**

Shift work results in sleep-wake disturbances, which cause sleepiness during night shifts and reduce sleep length and quality in daytime sleep after the night shift. In its serious form it is also called shift work sleep disorder. Various pharmacological products are used to ameliorate symptoms of sleepiness or poor sleep length and quality.

**OBJECTIVES:**

To evaluate the effects of pharmacological interventions to reduce sleepiness or to improve alertness at work and decrease sleep disturbances whilst off work, or both, in workers undertaking shift work.

**METHODS:**

*Search methods:* We searched CENTRAL, MEDLINE, EMBASE, PubMed and PsycINFO up to 20 September 2013 and ClinicalTrials.gov up to July 2013. We also screened reference lists of included trials and relevant reviews.

*Selection criteria:* We included all eligible randomised controlled trials (RCTs), including cross-over RCTs, of pharmacological products among workers who were engaged in shift work (including night shifts) in their present jobs and who may or may not have had sleep problems. Primary outcomes were sleep length and sleep quality while off work, alertness and sleepiness, or fatigue at work.

*Data collection and analysis:* Two authors independently selected studies, extracted data and assessed risk of bias in included trials. We performed meta-analyses where appropriate.

**MAIN RESULTS:**

We included 15 randomised placebo-controlled trials with 718 participants. Nine trials evaluated the effect of melatonin and two the effect of hypnotics for improving sleep problems. One trial assessed the effect of modafinil, two of armodafinil and one examined caffeine plus naps to decrease sleepiness or to increase alertness.

Melatonin (1 to 10 mg) after the night shift may increase sleep length during daytime sleep (mean difference, MD, 24 minutes, 95% confidence interval, CI, 9.8 to 38.9; seven trials, 263 participants, low quality evidence) and night-time sleep (MD 17 minutes, 95% CI 3.71 to 30.22; three trials, 234 participants, low quality evidence) compared to placebo. We did not find a dose-response effect. Melatonin may lead to similar sleep latency times as placebo (MD 0.37minutes, 95% CI - 1.55 to 2.29; five trials, 74 participants, low quality evidence).

Hypnotic medication, zopiclone, did not result in significantly longer daytime sleep length compared to placebo in one low quality trial and we could not use the data from the study on lormetazepam.

Armodafinil taken before the night shift probably reduces sleepiness by one point on the Karolinska Sleepiness Scale (KSS) (MD -0.99, 95% CI -1.32 to -0.67; range 1 to 10; two trials, 572 participants, moderate quality evidence) and increases alertness by 50 ms in a simple reaction time test (MD -50.0, 95% CI -85.5 to -15.5) at three months’ follow-up in shift work sleep disorder patients. Modafinil probably has similar effects on sleepiness (KSS) (MD -0.90, 95% CI -1.45 to -0.35; one trial, 183 participants, moderate quality evidence) and alertness in the psychomotor vigilance test in the same patient group. Post-marketing, severe skin reactions have been reported. Adverse effects reported by trial participants were headache, nausea and a rise in blood pressure. There were no trials in non-patient shift workers.

Based on one trial, caffeine plus pre-shift naps taken before the night shift decreased sleepiness (KSS) (MD -0.63, 95% CI -1.09 to -0.17).

We judged most trials to have a low risk of bias even though the randomisation method and allocation concealment were often not described.

**AUTHORS’ CONCLUSIONS:**

There is low quality evidence that melatonin improves sleep length after a night shift but not other sleep quality parameters. Both modafinil and armodafinil increase alertness and reduce sleepiness to some extent in employees who suffer from shift work sleep disorder but they are associated with adverse events. Caffeine plus naps reduces sleepiness during the night shift, but the quality of evidence is low. Based on one low quality trial, hypnotics did not improve sleep length and quality after a night shift.

We need more and better quality trials on the beneficial and adverse effects and costs of all pharmacological agents that induce sleep or promote alertness in shift workers both with and without a diagnosis of shift work sleep disorder. We also need systematic reviews of their adverse effects.

This is the abstract of a Cochrane Review published in the Cochrane Database of Systematic Reviews (CDSR) 2014, issue 8. Art. No.: CD009776. DOI: 10.1002/14651858.CD009776.pub2 (http://onlinelibrary.wiley.com/doi/10.1002/14651858.CD009776.pub2/pdf/abstract). For full citation and authors’ details see reference 1.
